# Methodological conduct of prognostic prediction models developed using machine learning in oncology: a systematic review

**DOI:** 10.1186/s12874-022-01577-x

**Published:** 2022-04-08

**Authors:** Paula Dhiman, Jie Ma, Constanza L. Andaur Navarro, Benjamin Speich, Garrett Bullock, Johanna A. A. Damen, Lotty Hooft, Shona Kirtley, Richard D. Riley, Ben Van Calster, Karel G. M. Moons, Gary S. Collins

**Affiliations:** 1 Centre for Statistics in Medicine, Nuffield Department of Orthopaedics, Rheumatology and Musculoskeletal Sciences, University of Oxford, Oxford, OX3 7LD UK; 2 NIHR Oxford Biomedical Research Centre, Oxford University Hospitals NHS Foundation Trust, Oxford, UK; 3 Julius Center for Health Sciences and Primary Care, University Medical Center Utrecht, Utrecht University, Utrecht, The Netherlands; 4 Cochrane Netherlands, University Medical Center Utrecht, Utrecht University, Utrecht, The Netherlands; 5 Basel Institute for Clinical Epidemiology and Biostatistics, Department of Clinical Research, University Hospital Basel, University of Basel, Basel, Switzerland; 6 Nuffield Department of Orthopaedics, Rheumatology, and Musculoskeletal Sciences, University of Oxford, Oxford, UK; 7 Centre for Prognosis Research, School of Medicine, Keele University, Staffordshire, ST5 5BG UK; 8 Department of Development and Regeneration, KU Leuven, Leuven, Belgium; 9 Department of Biomedical Data Sciences, Leiden University Medical Center, Leiden, the Netherlands; 10 EPI-centre, KU Leuven, Leuven, Belgium

**Keywords:** Prediction, Machine learning, Methodology

## Abstract

**Background:**

Describe and evaluate the methodological conduct of prognostic prediction models developed using machine learning methods in oncology.

**Methods:**

We conducted a systematic review in MEDLINE and Embase between 01/01/2019 and 05/09/2019, for studies developing a prognostic prediction model using machine learning methods in oncology. We used the Transparent Reporting of a multivariable prediction model for Individual Prognosis Or Diagnosis (TRIPOD) statement, Prediction model Risk Of Bias ASsessment Tool (PROBAST) and CHecklist for critical Appraisal and data extraction for systematic Reviews of prediction Modelling Studies (CHARMS) to assess the methodological conduct of included publications. Results were summarised by modelling type: regression-, non-regression-based and ensemble machine learning models.

**Results:**

Sixty-two publications met inclusion criteria developing 152 models across all publications. Forty-two models were regression-based, 71 were non-regression-based and 39 were ensemble models. A median of 647 individuals (IQR: 203 to 4059) and 195 events (IQR: 38 to 1269) were used for model development, and 553 individuals (IQR: 69 to 3069) and 50 events (IQR: 17.5 to 326.5) for model validation. A higher number of events per predictor was used for developing regression-based models (median: 8, IQR: 7.1 to 23.5), compared to alternative machine learning (median: 3.4, IQR: 1.1 to 19.1) and ensemble models (median: 1.7, IQR: 1.1 to 6). Sample size was rarely justified (*n* = 5/62; 8%). Some or all continuous predictors were categorised before modelling in 24 studies (39%). 46% (*n* = 24/62) of models reporting predictor selection before modelling used univariable analyses, and common method across all modelling types. Ten out of 24 models for time-to-event outcomes accounted for censoring (42%). A split sample approach was the most popular method for internal validation (*n* = 25/62, 40%). Calibration was reported in 11 studies. Less than half of models were reported or made available.

**Conclusions:**

The methodological conduct of machine learning based clinical prediction models is poor. Guidance is urgently needed, with increased awareness and education of minimum prediction modelling standards. Particular focus is needed on sample size estimation, development and validation analysis methods, and ensuring the model is available for independent validation, to improve quality of machine learning based clinical prediction models.

**Supplementary Information:**

The online version contains supplementary material available at 10.1186/s12874-022-01577-x.

## Background

Many medical decisions across all clinical specialties are informed by clinical prediction models [1–7], and they are often used in oncology, for example to assess risk of developing cancer, inform cancer diagnosis, predict cancer outcomes and prognosis, and guide treatment decisions [8–13]. Clinical prediction models use individual-level data, such as demographic information, clinical characteristics, and biomarker measurements, to estimate the individualised risk of existing or future clinical outcomes.

However, compared to the number of prediction models that are developed, very few are used in clinical practice and many models contribute to research waste [14–17]. This problem has been further exacerbated with the rapidly growing use of machine learning to develop clinical prediction models as a class of models perceived to provide automated diagnostic and prognostic risk estimation at scale. This has led to the production of a spiralling number of models to inform diagnosis and prognosis including in the field of oncology. Machine learning methods include neural networks, support vector machines and random forests.

Machine learning is often portrayed to offer more flexible modelling, the ability to analyse ‘big’, non-linear and high dimensional data, and modelling complex clinical scenarios [18, 19]. Despite this, machine learning methods are often applied to small and low dimensional settings [20, 21]. However, many perceived advantages of machine learning (over traditional statistical models like regression) to develop prediction models have not materialised into patient benefit. Indeed, many studies have found no additional performance benefit of machine learning over traditional statistical models [22–27].

A growing reason and concern resulting in their lack of implementation in clinical practice leading to patient benefit is the completeness of reporting, methodological quality and risk of bias in studies using machine learning methods [22, 25, 26, 28, 29]. Similarly, many regression-based prediction models have also not been implemented in clinical practice due to incomplete reporting and failure to follow methodological recommendations, often resulting in poor quality studies and models due to using sample sizes that are too small, risk of overfitting and lack of external validation of developed models [14, 30–35].

However, there is a lack of information about the methodological conduct of clinical prediction models developed using machine learning methods within oncology. We therefore aim to describe and evaluate the methodological conduct of clinical prediction models developed using machine learning in the field of oncology.

## Methods

We conducted a systematic search and review of prognostic model studies that use machine learning methods for model development, within the oncology clinical field. We excluded imaging and lab-based studies to focus on low dimensional, low signal and high noise clinical data settings. Machine learning was defined as a subset of artificial intelligence allowing for machines to learn from data with and without explicit programming.

The boundaries between machine learning and statistical, regression-based methods of prediction is often unclear and artificial, often seen as a cultural difference between methods and fields [36]. We therefore included studies that typically identify as machine learning, such as random forests and neural networks, and included any study in which the modelling method was declared as machine learning by authors of the included studies. For example, we included studies using logistic regression if they were explicitly labelled by the authors as machine learning, otherwise it was excluded.

### Protocol registration and reporting standards

This study is reported using the Preferred Reporting Items for Systematic Reviews and Meta-Analyses (PRISMA) guideline [37]. We registered this umbrella review with PROSPERO (ID: CRD42019140361) [38] that comprises of four distinct studies to evaluate (1) completeness of reporting, (2) risk of bias, (3) methodological conduct, and (4) spin over-interpretation.

### Information sources

We searched the MEDLINE (via OVID) and Embase (via OVID) medical literature databases for published clinical prediction modelling studies that use machine learning methods for model development, within the oncology clinical field. We searched for publications from 1 January 2019 to 5 September 2019, the date the searches were executed.

The search strategy comprised of three specific groups of search terms specific focussing on machine learning models, cancer, and prediction. Relevant Mesh and EMTREE headings were included as were free-text terms, searched in the title, abstract or keyword fields. We used general and specific machine learning model search terms such as “machine learning”, “deep learning”, “neural networks”, “random forest” or “support vector machine”. Cancer search terms included “cancer”, “tumour” or “malignancy”. General prediction and specific model performance search terms included “prediction”, “prognosis”, “discrimination”, “calibration” or “area under the curve”. The three specific groups of terms were combined with ‘AND’ to retrieve the final results set. The search was limited to retrieve studies published in 2019 only to ensure that a contemporary sample of studies were assessed in the review. The Embase search strategy was also limited to exclude conference abstract publications. No other limits were applied to the search and we also did not limit our search to specific machine learning methods so we could describe the types of models being used to develop prediction models in low dimensional setting and using clinical characteristics. Search strategies for both databases were developed with an information specialist (SK). The full search strategies for both included databases are provided in Supplementary Tables 1 and 2.

### Eligibility criteria

We included published studies developing a multivariable prognostic model using machine learning methods within oncology in 2019. A multivariable prognostic model was defined as a model that uses two or more predictors to produce an individualised predicted risk (probability) of a future outcome [39, 40]. We included studies predicting for any patient health-related outcome measurement (e.g., binary, ordinal, multinomial, time-to-event, continuous) and using any study design and data source (e.g., experimental studies such as randomised controlled trials, and observational studies such as prospective or retrospective cohort studies, case-control studies or studies using routinely collected data or e-health data).

We excluded studies that did not report the development of a multivariable prognostic model and studies that only validated models. We excluded diagnostic prediction model studies, speech recognition or voice pattern studies, genetic studies, molecular studies, and studies using imaging or speech parameters, or genetic or molecular markers as candidate predictors. Prognostic factor studies primarily focused on the association of (single) factors with the outcome were also excluded. Studies were restricted to the English language and to primary research studies only. Secondary research studies, such as reviews of prediction models, conference abstracts and brief reports, and preprints were excluded.

### Study selection, data extraction and management

All retrieved publications were imported into Endnote reference software where they were de-duplicated. Publications were then imported into Rayyan web application (www.rayyan.ai) where they were screened [41, 42].

Two independent researchers (PD, JM) screened the titles and abstracts of the identified publications. Two independent researchers, from a combination of five reviewers (PD, JM, GB, BS, CAN) reviewed the full text for potentially eligible publications and extracted data from eligible publications. One researcher screened and extracted from all publications (PD) and four researchers collectively screened and extracted from the same articles (JM, GB, BS, CAN). Disagreements were discussed and adjudicated by a sixth reviewer (GSC), where necessary.

To reduce subjectivity, the data extraction form to assess the methodological conduct was developed using formal and validated tools: the Transparent Reporting of a multivariable prediction model for Individual Prognosis Or Diagnosis (TRIPOD) guideline, the CHecklist for critical Appraisal and data extraction for systematic Reviews of prediction Modelling Studies (CHARMS) and the Prediction model Risk Of Bias ASsessment Tool (PROBAST) [39, 40, 43–45]. We then added specific machine learning items at the study design and analysis levels.

The form was piloted among all the five reviewers using five eligible publications [46]. Results of the pilot were discussed, and data extraction items were clarified amongst all reviewers to ensure consistent data extraction. All reviewers had expertise in the development, validation, and reviewing of prediction model studies using regression-based and machine learning methods. The data extraction form was implemented using Research Data Capture (REDCap) software [47].

### Data items

Descriptive data was extracted on the overall publication, including items for cancer type, study type, data source/study design, target population, type of prediction outcome, number and type of machine learning models used, setting, intended use and aim of the clinical prediction model. The TRIPOD, CHARMS and PROBAST guidance informed methodological items for extraction, including sample size calculation or justification, sampling procedure, blinding of the outcome and predictors, methods to address missing data, number of candidate predictors, model building strategies, methods to address censoring, internal validation methods and model performance measures (e.g. discrimination, calibration) [39, 40, 43–45].

Items for the results of each developed model were also extracted, including sample size (and number of events), and model discrimination and calibration performance results. For discrimination, we extracted the area under the receiver operating characteristic curve (AUC), i.e. the c-index (or c-statistic). For calibration, we extracted how this was evaluated (including whether the calibration slope and intercept were assessed), and whether a calibration plot with a calibration curve was presented. Items were extracted for the development and external validation (where available) of the models. We included additional items to capture specific issues associated with machine learning methods, such as methods to address class imbalance, data pre-processing, and hyperparameter tuning.

### Summary measures and synthesis of results

Findings were summarised using descriptive statistics and visual plots, alongside a narrative synthesis. Sample size was described using median, interquartile range (IQR) and range. The number of events reported in studies was combined with the reported number of candidate predictors to calculate the events per predictor. Analysis and synthesis of data was presented overall and by modelling type (regression-based, non-regression based and ensemble machine learning models). Ensemble models were defined models using a combination of different machine learning methods, including models where bagging or boosting was applied to a machine learning model (e.g., random forests, boosted random forests and boosted Cox regression). As we wanted to identify themes and trends in the methodological conduct of machine learning prediction models, we did not evaluate the nuances of each modelling approach and kept our evaluations at the study design and analysis levels.

Results for discrimination (AUC) and calibration (calibration slope and intercept) were summarised for the developed and validated machine learning models. Data was summarised for the apparent performance, internal validation performance, optimism-corrected performance, and the external validation performance.

All analyses were carried out in Stata v15 [48].

## Results

**Fig. 1:**
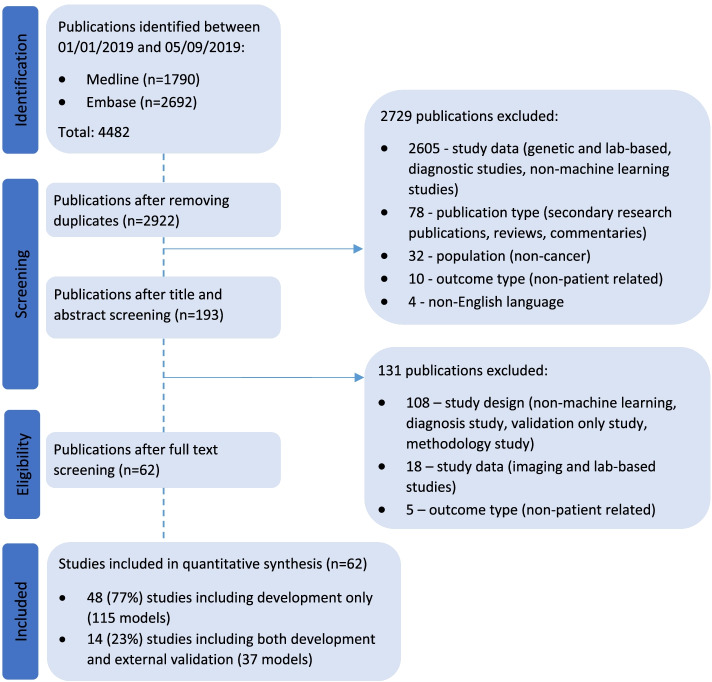
PRISMA flow diagram of studies included in the systematic review

Two thousand nine hundred twenty-two unique publications published between 1 January 2019 and 5 September 2019 were retrieved from MEDLINE and Embase. Title and abstract screening excluded 2729 publications and full text screening excluded a further 131 publications that did not meet the eligibility criteria. Sixty-two publications were included in our review, of which 77% (*n* = 48) were development only studies and 23% (*n* = 14) were development and external validation studies (Fig. 1). Study characteristics of included studies are presented in Supplementary Table 3.

### Model characteristics

A total of 152 prediction models were developed in the 62 publications. 115 (76%) models were from development-only studies and 37 (24%) were from development and validation studies. Overall, a median of two prediction models were developed per publication [range: 1–6] (Table 1). Classification trees (classification and regression trees and decision trees) (*n* = 28, 18%), logistic regression (*n* = 27, 18%), random forest (including random survival forest) (*n* = 23, 15%), neural networks (*n* = 18, 12%) and support vector machines (*n* = 12, 8%) were the most prevalent machine learning methods used. Thirty-nine models were developed using ensemble methods. Rationale for choice of machine learning method was provided for fewer than half of the models (*n* = 66/152, 43%).

**Table 1:** Model type of the 152 models developed in the 62 included publications

Model characteristics	All models (***n*** = 152)
	n (%)
**Regression-based models**	**42 (28)**
Logistic regression	26
Cox regression	7
Linear regression	3
LASSO (Logistic regression)	1
LASSO (Cox regression)	1
LASSO (model not specified)	3
Best subset regression with leave-out cross-validation	1
**Non-regression-based models**	**71 (47)**
Neural network (including deep learning)	18
Classification tree (e.g., CART, decision tree)	28
Support vector machine	12
Naive Bayes	6
K nearest neighbours	3
Other^a^	4
**Ensemble models**	**39 (26)**
Random forest (including random survival forest)	23
Gradient boosting machine	8
RUSBoost - boosted random forests	1
Bagging with J48 selected by Auto-WEKA	1
CoxBoost - boosted Cox regression	1
XGBoost: exTreme Gradient Boosting	1
Gradient boosting machine and Nystroem, combined using elastic net	1
Adaboost	1
Bagging, method not specified	1
Partitioning Around Medoid algorithm and complete linkage method	1
**Median number of models developed per study [IQR], range**	2 [1–4], 1–6

*CART* Classification And Regression Tree, *LASSO* Least Absolute Shrinkage and Selection Operator

^a^Other includes voted perceptron; fuzzy logic, soft set theory and soft set computing; hierarchical clustering model based on the unsupervised learning for survival data using the distance matrix of survival curves; Bayes point machine

### Study design features

#### Data source, sampling, treatment details and blinding

Models were mainly developed using registry data (*n* = 21/62, 34%) and validated using retrospective cohorts (*n* = 4/14, 29%). Consecutive sampling was specified in only eight studies (13%) [49–56], random sampling was used in one study [57] and one study sampled individuals by screening their entire database for eligible individuals [58]. For most studies, however, sampling methods were not reported (*n* = 52/62, 85%). Details of treatments received by patients at baseline were described during development in 53% of studies (*n* = 33/62), compared to 36% during validation (*n* = 5/14).

Blinding of predictor assessment to the outcome is needed to ensure predictors are not influenced by assessors and is especially important for predictors with subjective interpretation (e.g., patient reported outcome measures). However, only seven studies reported blinding predictor assessment to the outcome during model development (*n* = 7/62; 11%) [59–65] and two reported for model external validation (*n* = 2/62; 3%) [61, 63]. No studies reported blinding predictors assessment from other predictors during development and validation.

#### Candidate predictors and sample size

Nine studies provided rationale for their choice of candidate predictors (e.g., based on previous research) [60, 61, 63, 66–71] and one study forced a-priori predictors during model development [72] (Table 2). Fifty-six studies (90%) clearly reported their candidate predictors and a median of 16 candidate predictors were considered per study (IQR: 12 to 26, range: 4–33,788). Continuous candidate predictors were included in all studies, except one study for which it was unclear.

**Table 2:** Methods for predictor selection before and after modelling and hyperparameter tuning for 152 developed clinical prediction models, by modelling type

	All (***n*** = 152)	Regression-based models (***n*** = 42)	Non-regression-based models (***n*** = 71)	Ensemble models (***n*** = 39)
	n (%)	n (%)	n (%)	n (%)
**Predictor selection (before modelling) reported**	**52 (34)**	**20 (48)**	**23 (32)**	**9 (23)**
A-priori	5	3	1	1
No selection before modelling	3	1	2	–
Univariable	24	12	8	4
Clinically relevant and available data	1	–	1	–
Dropout technique at input layer	1	–	1	–
Random forest with RPA	9	1	6	2
Other modelling approach^a^	9	3	4	2
**Predictor selection (during modelling) reported**	**63 (41)**	**25 (59)**	**27 (38)**	**11 (28)**
Stepwise	6	4	2	–
Forward selection	6	5	–	1
Backward elimination	5	3	2	–
Full model approach (no selection)	11	4	5	2
Feed forward/backpropagation	5	–	5	–
Recursive partitioning analysis	7	–	7	–
LASSO	5	5	–	–
Gini index (minimised)	7	1	4	2
Cross validation	4	2	–	2
Other^b^	7	1	2	4
**Hyperparameter tuning methods reported**	**31 (21)**	**4 (10)**	**15 (23)**	**12 (31)**
Cross validation	19	4	7	8
Grid search (no further details provided)	6	–	4	2
Max tree depth	2	–	1	1
Adadelta method	2	–	2	–
Default software values	2	–	1	1

*RPA* Recursive partitioning analysis, *LASSO* Least Absolute Shrinkage and Selection Operator

^a^Modelling approaches include support vector machine, logistic regression, Cox regression, best subset linear regression, decision tree, meta-transformer (base algorithm of extra trees)

^b^Other includes change in unspecified performance measure, stochastic gradient descent, function, aggregation of bootstrapped decision trees and Waikato Environment for Knowledge Analysis for development-only studies, and hyperbolic tangent function, greedy algorithm for all models and using final chosen predictors from comparator model

Categorisation of continuous predictors results in a loss of information and is discouraged for prediction modelling research [73]. However, all continuous predictors were categorised before modelling for nearly a third of models from 24 studies (*n* = 44/152 models, 29%; *n* = 24/62 studies, 39%). For 35 models from 25 studies continuous predictors were implicitly categorised based on the modelling method used (e.g., random forests, CART) (*n* = 35/152 models, 23%; *n* = 25/62 studies, 40%).

A more acceptable approach to handle continuous predictors (for approaches that are not inherently based on categorisation as part of the method) is to assess the linearity assumption with the outcome and to model them non-linearly. Investigation into nonlinearity of predictors was explicitly reported in the methods for only two models (one study), a logistic regression model which included ‘interactions between variables and non-linearities’ and a support vector machine that included ‘different kernels (linear, polynomial and radial) and hyperparameters’ in its grid search to ‘fine tune the model’ [74]. For 33 models from 23 studies, nonlinearity of continuous predictors was considered implicit to modelling method used (e.g., neural networks, support vector machines and ensemble models), unless categorisation before modelling was specified (*n* = 33/152 models, 22%; *n* = 23/62 studies, 37%). A further eight models (three studies) also implicitly handled nonlinearity of continuous predictors in addition to some continuous predictors being categorised before modelling. For 28 models from 19 studies, continuous predictors were assumed to have a linear relationship with the outcome (*n* = 28/152 models, 18%; *n* = 19/62 studies, 31%). A further two models (one study) also categorised some predictors before modelling.

Methods to categorise predictors were also often unclear (*n* = 65/85, 80%). Methods for categorisation included clinically informed cut points (*n* = 3 studies) [6, 75, 76], percentiles (*n* = 4 studies) [6, 63, 70, 77], arbitrary dichotomisation (*n* = 3 studies) [63, 78, 79] and other data driven methods that included classification and regression trees, Monte Carlo simulation (authors report that ‘Monte Carlo simulation [was used] to evaluate multiple parameters by accounting for all possible dichotomous cut-offs and interactions between the inputted variables’) and fuzzification (*n* = 3 studies) [67, 80, 81].

Five studies calculated or provided rationale for their sample size for model development and were all based on flawed methodology [82]. This included, one study used 10 events per variable when developing a logistic regression model and a neural network [49], and another study used estimation of a relative hazard ratio between prognostic groups to calculate their sample size [83]. Two studies considered their sample size restricted by the size and availability of the existing data they were using (one randomised controlled trial [84] and one cohort study [66]) and one study justified sample size based on a time interval (e.g., consecutive adult patients over a 2-year period to allow a sufficient sample size for randomization to the training and validation data sets) [54]. One study reported traditional statistical sample size calculations are not applicable as ‘CART analysis generates nonparametric, predictive models’ [70]. Two studies calculated or provided rationale for their sample size for model validation. One study considered their sample size restricted by the size and availability of existing data they were using (randomised controlled trial [84]), and one study based their sample size on a power calculation (but details were not provided) [49].

Overall, a median of 647 individuals (IQR: 203 to 4059, range: 20 to 582,398) and 195 events (IQR: 38 to 1269, range: 7 to 45,979) was used for model development, and 553 individuals (IQR: 69 to 3069, range: 11 to 836,659) and 50 events (IQR: 17.5 to 326.5, range: 7 to 1323) for model validation. The study size informing model development was lower in development-only studies (median: 155 events, IQR: 38 to 392, range: 7 to 10,185), compared to development with validation studies (median: 872 events, IQR: 41.5 to 18,201, range: 22 to 45,797). A higher proportion of individuals with the outcome event were found in the development of regression-based models (median: 236 patients, IQR: 34 to 1326, range: 7 to 35,019), compared to non-regression-based machine learning (median: 62, IQR: 22 to 1075, range: 7 to 45,797) and ensemble models (median: 37, IQR: 22 to 241, range: 8 to 35,019) (Table 3).

**Table 3:** Sample size and number of candidate predictors informing analyses for 152 developed models, by modelling type

	Regression-based models (***n*** = 42)		Non-regression-based models (***n*** = 71)		Ensemble models (***n*** = 39)	
	Reported, n (%)	Median [IQR], range	Reported, n (%)	Median [IQR], range	Reported, n (%)	Median [IQR], range
**Total sample size**						
Model development	42 (100)	561 [203 to 2822], 20 to 582,398	70 (99)	447 [156 to 11,901], 20 to 582,398	39 (100)	768 [203 to 1599], 20 to 582,398
Internal validation^a^	20 (48)	122 [82 to 228], 47 to 291,200	35 (49)	145 [90 to 492], 47 to 291,200	24 (62)	162 [97 to 1510], 67 to 291,200
External validation	12 (29)	511 [67 to 2300], 11 to 836,659	14 (20)	793 [59 to 1675], 11 to 836,659	11 (28)	313 [229 to 836,659], 11 to 836,659
**Number of events**						
Model development	20 (48)	236 [34 to 1326], 7 to 35,019	37 (52)	62 [22 to 1075], 7 to 45,797	10 (26)	37 [22 to 241], 8 to 35,019
Internal validation^a^	2 (5)	41 [21 to 61], 21 to 61	3 (4)	61 [21 to 62], 21 to 62	1 (3)	61
External validation	8 (19)	81 [18 to 327], 7 to 513	11 (15)	19 [7 to 513], 7 to 1323	5 (13)	81 [81 to 81], 7 to 513
**No. candidate predictors**	38 (90)	21 [15 to 34], 6 to 33,788	64 (90)	16 [12 to 25], 5 to 33,788	36 (92)	25 [14 to 37], 4 to 33,788
**Events per predictor**^b^	20 (48)	8.0 [7.1 to 23.5], 0.2 to 5836.5	35 (49)	3.4 [1.1 to 19.1], 0.2 to 5836.5	10 (26)	1.7 [1.1 to 6.0], 0.7 to 5836.5

^a^Combines all internal validation methods, e.g., split sample, cross validation, bootstrapping

^b^Events per predictor for model development

Combining the number of candidate predictors with number of events used for model development, a median 7.4 events were available per predictor (IQR: 1.7 to 15.2, range: 0.2 to 153.6) for development only studies and 49.2 events per predictor for development with validation studies (IQR: 2.9 to 2939.1, range 1.0 to 5836.5). A higher number of events per predictor was used for developing regression-based models (median: 8, IQR: 7.1 to 23.5, range: 0.2 to 5836.5), compared to alternative machine learning (median: 3.4, IQR: 1.1 to 19.1, range: 0.2 to 5836.5) and ensemble models (median: 1.7, IQR: 1.1 to 6, range: 0.7 to 5836.5). The distribution of the events per predictor, by modelling type, is provided in Supplementary Figs. 1 and 2.

#### Validation procedures

When internally validating a prediction model, using the random split sample is not efficient use of the available data as it reduces the sample size available for developing the prediction model more robustly [39, 44]. However, a split sample approach was the most popular method to internally validate the developed models (*n* = 25/62, 40%).

Resampling methods, such cross-validation and bootstrapping are preferred approaches as they use all the data for model development and internal validation [39, 44]. Bootstrapping was used in seven studies (11%) [61, 63, 77, 79, 85–87] and cross-validation in 15 studies (24%) [49–51, 53, 57, 71, 74, 76, 88–94]. Four studies used a combination of approaches; one study used split sample and bootstrapping [95], two studies used split sample and cross-validation [64, 96], and one study used cross-validation and bootstrapping [97]. For 11 studies, internal validation methods were unclear (18%) [65, 70, 75, 80, 83, 84, 98–102].

Of the 14 development with validation (external) studies, two used geographical validation [49, 90], three used temporal validation [63, 71, 103] and 9 used independent data that was geographically and temporally different from the development data to validate their models [58, 61, 69, 75, 80, 84, 86, 93, 95]. Seven studies (50%) reported differences and similarities in definitions between the development and validation data [58, 61, 69, 71, 75, 84, 90].

### Analysis methods

#### Missing data and censoring

Handling of missing data was poor. The assumed mechanism for missingness was not reported in any study. Using a complete case analysis to handle missing data, not only reduces the amount of data available to develop the prediction model but may also lead to biased results with an unrepresentative sample of the target population [104–106]. However, nearly half of studies performed a complete case analysis (*n* = 30/62, 48%), of which 87% of studies (*n* = 26/30, 87%) excluded missing data (outcome or predictor) as part of study eligibility criteria. For 12 of the studies reporting the amount of missing data excluded as part of the study eligibility criteria (*n* = 12/62, 19%), a median of 11.1% (IQR: [4.0–27.9], range: 0.5–57.8) of individuals were excluded from the data prior to analysis [65, 71, 76–78, 81, 83, 89, 99, 102, 107, 108].

For six studies (*n* = 6/62, 10%), mean, median, or mode imputation was used (for three studies this was in addition to exclusion of missing data as part of the study eligibility criteria) [51, 56, 58, 76, 102, 108]. For five studies (*n* = 5/30, 17%) multiple imputation was used (of which one was used in addition to exclusion of missing data as part of the study eligibility criteria) [50, 60, 66, 96, 107], including one study using missForest imputation [96]. Procedure methods for multiple imputation was not appropriately described. An imputation threshold was specified in two studies, which only imputed data if missing data was less than 25 and 30%, respectively [60, 96]. One study specified the number of repetitions for the multiple imputation [50]. Two studies used subsequent follow up data and another study used a k-nearest neighbour algorithm [65, 95].

Missing data in the development data was presented by all or some candidate predictors in 13 studies (*n* = 13/62, 20%). Two studies (out of 14) presented missing data for all predictors during validation.

Information regarding loss to follow up and censoring was rarely reported. Only 14 studies explicitly mentioned methods to handle loss to follow-up (*n* = 14/62 studies, 17%), of which six studies excluded patients that were lost to follow up [63, 77, 86, 89, 98, 107], and one study reported that the ‘definition of treatment failure does not capture patients lost to follow-up due to future treatments at other institutions or due to the cessation of treatment for other reasons’ [56]. For the remaining seven studies, patients who were lost of follow up were included in the study and outcome definition [53, 65, 67, 83, 90, 100, 109]. For example, Hammer et al. reported that ‘if no event of interest had occurred, patients were censored at the time of last documented contact with the hospital’ [83].

Eleven studies developed 24 models for a time to event outcome(*n* = 11/62 studies, 18%; *n* = 24/152 models, 16%); these were seven Cox regression models, one logistic regression model, one linear regression model, two neural networks, three random forests (including two random survival forests), four gradient boosting machines, one decision tree, two naïve bayes algorithms, one hierarchical clustering model based on the unsupervised learning for survival data using the distance matrix of survival curves, and two ensemble models (CoxBoost and Partitioning Around Medoid algorithm). Of these, only 10 models explicitly accounted for censored observations (*n* = 10/24, 42%).

#### Data pre-processing, class imbalance

Only two studies assess collinearity between predictors (3%) [65, 69]. Nine studies used data pre-processing techniques. One study reduced data variables using automated feature selection [56] and seven studies transformed and/or standardised their predictors (including normalisation) [49, 57, 58, 84, 92, 95, 110] . One used one-hot coding to transform categorical data and create dummy predictors in addition to predictor standardisation [58]. One study inappropriately used propensity score to obtain comparable matched groups between events and non-events [111].

Class imbalance was examined in 19 models (from six development-only studies). One study used Synthetic Minority Oversampling TEchnique (SMOTE) to generate synthetic samples on the minority (positive) class using K-nearest neighbourhood graph [88], another study also used oversampling on the minority (dead) class to balance the number of ‘alive and ‘dead’ cases [107]. Undersampling was used in two studies [92, 102]. For two studies, methods to address class imbalance was unclear and only described ‘addressing class imbalance during hyperparameter tuning’ [72] and using ‘5-fold cross validation’ [51]. The four studies using oversampling and undersampling methods to address class imbalance failed to then examine calibration or recalibrate their models which would be miscalibrated given the artificial event rate created using these approaches.

#### Predictor selection, model building and hyperparameter tuning

Univariable and multivariable predictor selection before model building can lead to biased results, incorrect predictor selection for modelling and increased uncertainty in model structure [112–115]. However, methods for predictor selection before modelling were not reported for 66% of models (*n* = 100/152), and of the 52 models that did report predictor selection before modelling, 24 used univariable screening selection to select predictors (46%), and for 18 models, predictors were selected before modelling by using other modelling approaches (35%), for example a multivariable logistic regression was developed, and predictors retained in this model were then entered into a random forest.

Methods for predictor selection during modelling were reported for 41% of developed models (*n* = 63/152). Forward selection, backward elimination and stepwise methods were most commonly used (*n* = 17/63, 27%) and were predominantly for regression-based machine learning models, with only five non-regression machine learning and ensemble model using them. Seven non-regression machine learning models used recursive partitioning and seven models (overall) were based on minimising the Gini index (13%). Only seven models (three regression based, three non-regression based machine learning models and one ensemble model) explicitly planned assessment of interactions [65, 74, 80, 93].

Thirty-two models reported hyperparameter tuning methods. Most of these models (*n* = 19/32, 59%) used cross-validation (14 used k-fold, two used repeated k-fold and for three models it cross-validation type was unclear), including four regression-based machine learning models. Six non-regression machine learning and ensemble models used grid search for hyperparameter tuning but did not provide any further details (e.g., one study stated that ‘an extensive grid search was applied to find the parameters that could best predict complications in the training sample’ [78]).

### Model performance

Overall fit of the developed model was reported for three studies (two used the Brier Score and one used R-squared). Model discrimination was reported in 76% (*n* = 47/62) of all studies. Discrimination (i.e., c-statistic, c-index) was reported in all studies predicting a binary or time-to-event (survival) outcome. Three studies predicting a time-to-event outcome (*n* = 11 models) incorrectly calculated discrimination and used an approach which does not account for censored observations. The root mean square log error was reported for the one study predicting a continuous (length of stay) outcome.

Model calibration was only reported in 18% (*n* = 11/62) of studies. Of these, 10 studies presented a calibration plot, including four studies that also reported estimates of the calibration slope and intercept. One study reported the Hosmer Lemeshow test, which is widely discouraged as a measure of calibration as it provides no assessment of the direction or magnitude of any miscalibration [39].

Of the 11 studies reporting calibration, three studies modelled for a time to event outcome. One study presented 3- and 5-year survival calibration plots [86], one study presented a linear regression and plot of the predicted and actual survival time [52], and one study presented a 1-year calibration plot [77].

Other performance measures were reported in 69% of studies (*n* = 43/62), which predominantly included classification measures such as sensitivity, specificity, accuracy, precision and F1 score (*n* = 35/43, 81%). For these classification measures, seven reported the associated cut-off values.

Three studies reported results of a net benefit and decision curve analysis, and one study reported the net reclassification index and integrated discrimination improvement. Measures of error were reported in four studies and included mean per class error; absolute relative error, percentage difference between observed and predicted outcomes; root node error; applied root mean square error.

#### Model performance results

Apparent discrimination (AUC) was reported for 89 models (*n* = 89/152, 59%), optimism corrected AUC was reported for 26 models (*n* = 26/152, 17%) and external validation AUC results were reported for 26 models (*n* = 26/37, 70%). The median apparent AUC was 0.75 (IQR: 0.69–0.85, range: 0.54–0.99), optimism corrected AUC was 0.79 (IQR: 0.74–0.85, range: 0.56–0.93), and validation AUC was 0.73 (IQR: 0.70–0.78, range: 0.51–0.88).

Both apparent and optimism corrected AUC was reported for eight models, in which we found a median 0.05 reduction in AUC (IQR: −0.09 to −0.03, range: −0.14 to 0.005). Both apparent and validation AUC was reported for 11 models, in which we found a median 0.02 reduction in AUC (IQR: −0.04 to −0.002, range: −0.08 to 0.01).

### Risk groups and model presentation

Risk groups were explicitly created in four studies, of which three provided cut-off boundaries for the risk groups. Two studies created 3 groups, one created 4 groups and one created 5 groups. To create the risk groups, three studies used data driven methods including one study that used a classification and regression tree, and for one study it was unclear.

Two development with validation studies created risk groups, both provided cut-off boundaries and created 3 groups. To create the risk groups, one study used data driven methods and for the other it was unclear.

Presentation or explanation of how to use the prediction model (e.g., formula, decision tree, calculator, code) was reported in less than half of studies (*n* = 28/62, 45%) of studies. Presentation of the full (final) regression-based machine learning model was provided in two studies (*n* = 2/28, 7%) [61, 87]. Decision trees (including CART) were provided in 14 studies (*n* = 14/28, 50%). Code or a link or reference to a web calculator was provided in six studies (*n* = 6/28, 21%), and a point scoring system or nomogram was provided in four studies (*n* = 4/28, 14%). Two studies provided a combination of a point scoring system or code, with a decision tree (*n* = 2/28, 7%). Thirty-six studies (*n* = 36/62, 58%) developed more than 2 prediction models, and a the ‘best’ model was identified in 30 studies (*n* = 30/36, 83%). Twenty-eight studies identified the ‘best’ model based on model performance measures (i.e., AUC, net benefit, and classification measures), one study model based it on model parsimony, and for one study it was unclear.

## Discussion

### Summary of findings

In this review we assessed the methodological conduct of studies developing author defined machine learning based clinical prediction models in the field of oncology. Over a quarter of statistical regression models were considered machine learning. We not only found poor methodological conduct for nearly all developed and validated machine learning based clinical prediction models, but also a large amount of heterogeneity in the choice of model development and validation methodology, including the choice of modelling method, sample size, model performance measures and reporting.

A key factor contributing to the poor quality of these models was unjustified, small sample sizes used to develop the models. Despite using existing data from electronic health records and registries, most models were informed by small datasets with too few events. Non-regression-based machine learning and ensemble models were developed using smaller datasets (lower events per predictor), compared to regression-based machine learning models. Use of smaller datasets for non-regression and ensemble machine learning models is problematic and increases their risk of overfitting further due to increased flexibility and categorisation of prediction inherent to many machine learning methods [116, 117].

The risk of overfitting in the included studies and models was further exacerbated by split sample internal validation approaches, exclusion of missing data, univariable predictor selection before model building and stepwise predictor selection during model building. Few models also appropriately handled complexities in the data, for example, methods for censoring were not reported in many studies and was rarely accounted for in models developed for a time for event outcome.

Model performance measures were often discrimination and classification performance measures and were not corrected for optimism, yet these measures were often used to identify the ‘best’ model in studies developing and comparing more than one model. Under and over sampling methods were used to overcome class imbalance, however this results in distortion of the outcome event rate resulting in poorly calibrated models.; however, calibration was rarely reported in studies.

Over half the developed models would not be able to be independently validated, an important step for implementation of prediction models in clinical practice, as they were not reported or available (via code or web calculator) in their respective studies.

### Literature

Our review supports evidence of poor methodological quality of machine learning clinical prediction models which has been highlighted by cancer and non-cancer reviews [22, 26, 118, 119]. Methodological shortcomings have also been found in prediction modelling reviews focussed on only regression-based cancer prediction models. Our findings are comparable to these reviews which highlight inappropriate use of methods and lack of sufficient sample size for development and external validation of prediction models [120–123].

Li et al. reviewed machine learning prediction models for 5-year breast cancer survival and compared machine learning to statistical regression models [118]. They found negligible improvement in the performance of machine learning models and highlighted low sample sizes, lack of pre-processing steps and validation methods and problematic areas for these models. Christodoulou et al. conducted a systematic review of studies comparing machine learning models to logistic regression and also found inconclusive evidence of superiority of machine learning over logistic regression, a low quality or indeed high risk of bias associated to model and a need to further reporting and methodological guidance [22].

Insufficient sample size when developing and validating machine learning based clinical prediction models is a common methodological flaw in studies [22, 23, 26]. However, it may be a bigger problem for machine learning models with lower events per variable observed, compared to regression-based models and studies have shown that much larger sample sizes are needed when using machine learning methods and so the impact and risk of bias introduced from these insufficient sample sizes may be much larger [117, 124].

### Strengths and limitations

This review highlights the common methodological flaws found in studies developing machine learning based clinical prediction models in oncology. Many existing systematic reviews have focussed on the quality of models in certain clinical sub-specialties and cancer types, and we provide a broader view and assessment that focusses on the conduct of clinical prediction model studies using machine learning methods in oncology.

We calculate the event per predictor, instead of the events per predictor parameter as the number of predictor parameters was not possible to ascertain due to the ‘black box’ nature of machine learning models. This means that the sample size may be more inadequate than is highlighted in our review.

Though we searched MEDLINE and Embase, two major information databases for studies that developed (and validated) a machine learning based clinical prediction model, we may have missed eligible publications. Our studies are also restricted to models that were published during 01 Jan 2019 and 05 Sept 2019 resulting in missing models published since our search date. However, our aim for this review was to describe a contemporary sample of publications to reflect current practice. Further, as our findings agree with the existing evidence, it is unlikely that additional studies would change the conclusion of this review.

We included a study by Alcantud et al. [81] which used fuzzy and soft set theory, traditionally an artificial intelligence method that resembles human knowledge and reasoning, as opposed to a machine learning method that learns from data. This was a result of using a broader search string to describe the types of models being used to develop prediction models in low dimensional setting and using clinical characteristics. Removing this study from our review does not change our findings and conclusions.

### Future research

Methodological guidance, better education, and increased awareness on the minimum scientific standards for prediction modelling research is urgently needed to improve the quality and conduct of machine learning models. The Transparent Reporting of a multivariable prediction model for Individual Prognosis Or Diagnosis (TRIPOD) collaboration has initiated the development of a TRIPOD statement and PROBAST quality assessment tool specific to machine learning (TRIPOD-AI and PROBAST-AI) to improve reporting conduct and evaluation of these models [39, 125]. Both this review and a sister review of diagnostic and prognostic models have been conducted to inform these guidelines (PROSPERO ID: CRD42019161764).

These guidelines need to be complemented with methodological guidance to support researchers developing clinical prediction models using machine learning to ensure use better and efficient modelling methods. There is a primary need for sample size guidance that will ensure informed and justified use of data and machine learning methods to develop these models.

Development of machine learning based clinical prediction models in general and in oncology is rapid. Periodic reviews and re-reviews are needed so evidence reflects current practice. These reviews should both focus on individual clinical domains and be cancer specific but should also focus on machine learning based clinical prediction models.

## Conclusions

The methodological conduct of machine learning based clinical prediction models is poor. Reporting and methodological guidance is urgently needed, with increased awareness and education of minimum prediction modelling scientific standards. A particular focus is needed on sample size estimation, development and validation analysis methods, and ensuring the developed model is available for independent validation, to improve quality of machine learning based clinical prediction models.

## Supplementary Information

**Additional file 1.**

## Data Availability

The datasets generated and/or analysed during the current study are available in the Open Science Framework repository (https://osf.io/3aezj/).

## Abbreviations

AUC: Area under the curve
TRIPOD: The Transparent Reporting of a multivariable prediction model for Individual Prognosis Or Diagnosis
PROBAST: Prediction model Risk Of Bias ASsessment Tool
CART: Classification and regression tree
RPA: Recursive partitioning analysis
LASSO: Least Absolute Shrinkage and Selection Operator
CHARMS: CHecklist for critical Appraisal and data extraction for systematic Reviews of prediction Modelling Studies
PRISMA: Preferred Reporting Items for Systematic Reviews and Meta-Analyses guideline
IQR: Interquartile range
