# Supplementary material for: Methodological conduct of prognostic prediction models developed using machine learning in oncology: a systematic review
Source: BMC Med Res Methodol. 2022 Apr 8;22:101. doi: 10.1186/s12874-022-01577-x (PMC8991704; doi:10.1186/s12874-022-01577-x)
Supplement: Supplementary file 1 — Additional file 1. [file 12874_2022_1577_MOESM1_ESM.docx]

**Supplementary information**

**Supplementary table 1 - MEDLINE search strategy**

Database and platform: MEDLINE (Ovid MEDLINE® Epub Ahead of Print, In-Process & Other Non-Indexed Citations, Ovid MEDLINE® Daily and Ovid MEDLINE®) 1946 to present (via Ovid)

Publication date limit: 2019 only

Search date: 5 September 2019

| 1 | Machine Learning/ |
| --- | --- |
| 2 | (machine adj1 (learn$ or model$)).ti,ab,kw. |
| 3 | Deep Learning/ |
| 4 | (deep adj2 learn$).ti,ab,kw. |
| 5 | exp Supervised Machine Learning/ |
| 6 | (supervised adj2 machine adj2 learn$).ti,ab,kw. |
| 7 | ((support or relevance) adj2 vector adj2 (machine$ or classification$)).ti,ab,kw. |
| 8 | "Neural Networks (Computer)"/ |
| 9 | (neural adj2 network$).ti,ab,kw. |
| 10 | ((statistical or "statistical-learning") adj1 (learn$ or strateg$)).ti,ab.kw. |
| 11 | (multi adj2 layer adj1 perceptron$).ti,ab,kw. |
| 12 | (random adj2 forest$).ti,ab,kw. |
| 13 | "RF classifi$".ti,ab,kw. |
| 14 | (lasso or ridge or kernel or ensemble or bagging or bagged or bootstrap$ or boosting or boosted or fuzzy).ti,ab,kw. |
| 15 | ((penali?ed or regulari?ed) adj2 ('likelihood' or 'regression' or 'logistic' or 'survival' or 'estimat$' or 'function$' or 'method$' or 'least' or 'ensemble')).ti,ab,kw. |
| 16 | ((classification or regression or estimation or decision) adj2 tree$).ti,ab,kw. |
| 17 | (bayes$ adj1 network$).ti,ab,kw. |
| 18 | (nearest adj1 neighbo?r).ti,ab,kw. |
| 19 | (k-nearest adj1 neighbo?r).ti,ab,kw. |
| 20 | (elastic adj1 net).ti,ab,kw. |
| 21 | (naive adj1 bayes$).ti,ab,kw. |
| 22 | ((nonparametric or "non-parametric") adj2 (model$ or analys$)).ti,ab,kw |
| 23 | (KNN or ANN or ANNs or RNN or RF or SVM or NB or CART or DT or MLP).ti,ab,kw. |
| 24 | or/1-23 |
| 25 | Logistic Models/ |
| 26 | (logistic adj2 (model$ or regression)).ti,ab,kw. |
| 27 | Linear Models/ |
| 28 | (linear adj2 (model$ or regression)).ti,ab,kw. |
| 29 | (proportion$ adj2 odds adj2 regression).ti,ab,kw. |
| 30 | Least-Squares Analysis/ |
| 31 | (least adj2 square$).ti,ab,kw. |
| 32 | Survival Analysis/ |
| 33 | (survival adj1 (analys$ or model$)).ti,ab,kw. |
| 34 | Proportional Hazards Models/ |
| 35 | (proportional adj1 hazard$).ti,ab,kw. |
| 36 | ((cox or parametric) adj1 (regression or model$)).ti,ab,kw. |
| 37 | (semi adj2 parametric adj1 (regression or model$)).ti,ab,kw. |
| 38 | Disease-Free Survival/ |
| 39 | Progression-Free Survival/ |
| 40 | ((disease or progression or event) adj2 free adj1 survival).ti,ab,kw. |
| 41 | (overall adj1 survival).ti,ab,kw. |
| 42 | or/25-41 |
| 43 | 24 and 42 |
| 44 | 24 or 43 |
| 45 | exp Neoplasms/ |
| 46 | (cancer$ or tumour$ or tumor$ or carcinoma$ or malignan$ or neoplas$ or sarcoma$ or adenocarcinoma$ or carcinogen$ or metasta$ or oncolog$).ti,ab,kw. |
| 47 | or/45-46 |
| 48 | Prognosis/ |
| 49 | (prognos$ adj1 (modelling or modeling or model or models or predict$ or index or performance or nomogram or tools or ability or accuracy or probability or risk or factor$ or marker$ or biomarker$)).ti,ab,kw. |
| 50 | "risk model$".ti,ab,kw. |
| 51 | "predict$ the prognosis of".ti,ab,kw. |
| 52 | "predict$ the risk of".ti,ab,kw. |
| 53 | "predict$ the probability of".ti,ab,kw. |
| 54 | Probability/ |
| 55 | (probability adj1 (modelling or modeling or model or models)).ti,ab,kw. |
| 56 | (predict$ adj1 (modelling or modeling or model or models or nomogram or tools or performance or ability or index or accuracy or probability or risk or factor$ or marker$ or biomarker$)).ti,ab,kw. |
| 57 | "candidate predictor$".ti,ab,kw. |
| 58 | "predictive clinical parameter$".ti,ab,kw. |
| 59 | ((discrimination or discriminative or discriminatory) adj1 (accuracy or ability or performance or value or model or models or power or capacity or capabilit$ or efficiency)).ti,ab,kw. |
| 60 | (discriminability or c-index or c-statistic or concordance or DCA).ti,ab,kw. |
| 61 | "decision curve".ti,ab,kw. |
| 62 | (calibrat$ adj1 (plot$ or curve$ or slope$ or model or models)).ti,ab,kw. |
| 63 | (brier adj1 score$).ti,ab,kw. |
| 64 | (performance adj1 (classification or classifier or clinical or accuracy or validation or metrics or diagnostic or AUC)).ti,ab,kw. |
| 65 | (sensitivity or specificity or PPV or NPV).ti,ab,kw. |
| 66 | "correctly classified".ti,ab,kw. |
| 67 | "clinical accuracy".ti,ab,kw. |
| 68 | "positive predictive value$".ti,ab,kw. |
| 69 | "negative predictive value$".ti,ab,kw. |
| 70 | (classification or classifier).ti,ab,kw. |
| 71 | Area Under Curve/ |
| 72 | "Area under the curve".ti,ab,kw. |
| 73 | "Area under the ROC curve".ti,ab,kw. |
| 74 | "Area under the ROC".ti,ab,kw. |
| 75 | "Area Under the Receiver Operat$ Characteristic$".ti,ab,kw. |
| 76 | ROC Curve/ |
| 77 | "receiver operating characteristic$".ti,ab,kw. |
| 78 | (ROC or AUC or AUROC).ti,ab,kw. |
| 79 | "Hosmer-Lemeshow".ti,ab,kw. |
| 80 | "H-L test".ti,ab,kw. |
| 81 | "expected ratio".ti,ab,kw. |
| 82 | "observed ratio".ti,ab,kw. |
| 83 | "E:O ratio".ti,ab,kw. |
| 84 | or/48-83 |
| 85 | 44 and 47 and 84 |
| 86 | Limit 85 to yr="2019" |

**Supplementary table 2 - EMBASE search strategy**

Database and platform: Embase 1974 to present (via Ovid)

Publication date limit: 2019 only

Search date: 5 September 2019

| 1 | exp Machine Learning/ |
| --- | --- |
| 2 | (machine adj1 (learn$ or model$)).ti,ab,kw. |
| 3 | (deep adj2 learn$).ti,ab,kw. |
| 4 | (supervised adj2 machine adj2 learn$).ti,ab,kw. |
| 5 | ((support or relevance) adj2 vector adj2 (machine$ or classification$)).ti,ab,kw. |
| 6 | (neural adj2 network$).ti,ab,kw. |
| 7 | ((statistical or "statistical-learning") adj1 (learn$ or strateg$)).ti,ab,kw. |
| 8 | (multi adj2 layer adj1 perceptron$).ti,ab,kw. |
| 9 | (random adj2 forest$).ti,ab,kw. |
| 10 | "RF classifi$".ti,ab,kw. |
| 11 | Bootstrapping/ |
| 12 | (lasso or ridge or kernel or ensemble or bagging or bagged or bootstrap$ or boosting or boosted or fuzzy).ti,ab,kw. |
| 13 | ((penali?ed or regulari?ed) adj2 (likelihood or regression or logistic or survival or estimat$ or function$ or method$ or least or ensemble)).ti,ab,kw. |
| 14 | Decision Tree/ |
| 15 | ((classification or regression or estimation or decision) adj2 tree$).ti,ab,kw. |
| 16 | (naive adj1 bayes$).ti,ab,kw. |
| 17 | (bayes$ adj1 network$).ti,ab,kw. |
| 18 | (nearest adj1 neighbo?r).ti,ab,kw. |
| 19 | (k-nearest adj1 neighbo?r).ti,ab,kw. |
| 20 | (elastic adj1 net).ti,ab,kw. |
| 21 | Nonparametric Test/ |
| 22 | ((nonparametric or "non-parametric") adj2 (model$ or analys$)).ti,ab,kw. |
| 23 | (KNN or ANN or ANNs or RNN or RF or SVM or NB or CART or DT or MLP).ti,ab,kw. |
| 24 | or/1-23 |
| 25 | Logistic Regression Analysis/ |
| 26 | (logistic adj2 (model$ or regression)).ti,ab,kw. |
| 27 | Linear Regression Analysis/ |
| 28 | (linear adj2 (model$ or regression)).ti,ab,kw. |
| 29 | (proportion$ adj2 odds adj2 regression).ti,ab,kw. |
| 30 | Least Square Analysis/ |
| 31 | (least adj2 square$).ti,ab,kw. |
| 32 | Survival Analysis/ |
| 33 | (survival adj1 (analys$ or model$)).ti,ab,kw. |
| 34 | Proportional Hazards Models/ |
| 35 | (proportional adj1 hazard$).ti,ab,kw. |
| 36 | ((cox or parametric) adj1 (regression or model$)).ti,ab,kw. |
| 37 | (semi adj2 parametric adj1 (regression or model$)).ti,ab,kw. |
| 38 | Disease-Free Survival/ |
| 39 | Progression-Free Survival/ |
| 40 | ((disease or progression or event) adj2 free adj1 survival).ti,ab,kw. |
| 41 | (overall adj1 survival).ti,ab,kw. |
| 42 | or/25-41 |
| 43 | 24 and 42 |
| 44 | 24 or 43 |
| 45 | exp Neoplasm/ |
| 46 | (cancer$ or tumour$ or tumor$ or carcinoma$ or malignan$ or neoplas$ or sarcoma$ or adenocarcinoma$ or carcinogen$ or metasta$ or oncolog$).ti,ab,kw. |
| 47 | or/45-46 |
| 48 | exp Prognosis/ |
| 49 | (prognos$ adj1 (modelling or modeling or model or models or predict$ or index or performance or nomogram or tools or ability or accuracy or probability or risk or factor$ or marker$ or biomarker$ or parameter$)).ti,ab,kw. |
| 50 | "risk model$".ti,ab,kw. |
| 51 | "predict$ the prognosis of".ti,ab,kw. |
| 52 | "predict$ the risk of".ti,ab,kw. |
| 53 | "predict$ the probability of".ti,ab,kw. |
| 54 | (probability adj1 (modelling or modeling or model or models)).ti,ab,kw. |
| 55 | Prediction/ |
| 56 | (predict$ adj1 (modelling or modeling or model or models or nomogram or tools or performance or ability or index or accuracy or probability or risk or factor$ or marker$ or biomarker$)).ti,ab,kw. |
| 57 | "candidate predictor$".ti,ab,kw. |
| 58 | "predictive clinical parameter$".ti,ab,kw. |
| 59 | ((discrimination or discriminative or discriminatory) adj1 (accuracy or ability or performance or value or model or models or power or capacity or capabilit$ or efficiency)).ti,ab,kw. |
| 60 | (discriminability or c-index or c-statistic or concordance or DCA).ti,ab,kw. |
| 61 | "decision curve".ti,ab,kw. |
| 62 | Calibration/ |
| 63 | (calibrat$ adj1 (plot$ or curve$ or slope$ or model or models)).ti,ab,kw. |
| 64 | (brier adj1 score$).ti,ab,kw. |
| 65 | (performance adj1 (classification or classifier or clinical or accuracy or validation or metrics or diagnostic or AUC)).ti,ab,kw. |
| 66 | Validation Process/ |
| 67 | (sensitivity or specificity or PPV or NPV).ti,ab,kw. |
| 68 | "correctly classified".ti,ab,kw. |
| 69 | (classification or classifier).ti,ab,kw. |
| 70 | "clinical accuracy".ti,ab,kw. |
| 71 | "positive predictive value$".ti,ab,kw. |
| 72 | "negative predictive value$".ti,ab,kw. |
| 73 | Predictive value/ |
| 74 | Probability/ |
| 75 | "Area Under the Curve"/ |
| 76 | "Area Under the Curve Ratio"/ |
| 77 | "Area under the curve".ti,ab,kw. |
| 78 | "Area under the ROC curve".ti,ab,kw. |
| 79 | "Area under the ROC".ti,ab,kw. |
| 80 | "Area Under the Receiver Operat$ Characteristic$".ti,ab,kw. |
| 81 | ROC Curve/ |
| 82 | Receiver Operating Characteristic/ |
| 83 | "receiver operating characteristic$".ti,ab,kw. |
| 84 | (ROC or AUC or AUROC).ti,ab,kw. |
| 85 | "Hosmer-Lemeshow".ti,ab,kw. |
| 86 | "H-L test".ti,ab,kw. |
| 87 | "expected ratio".ti,ab,kw. |
| 88 | "observed ratio".ti,ab,kw. |
| 89 | "E:O ratio".ti,ab,kw. |
| 90 | or/48-89 |
| 91 | 44 and 47 and 90 |
| 92 | conference abstract.pt. |
| 93 | conference abstract.st. |
| 94 | 92 or 93 |
| 95 | 91 not 94 |
| 96 | Limit 95 to yr="2019" |

**Supplementary table 3.** Study and design characteristics of the 62 included publications, by study type.

|  | **All (n=62)** | **Development only (n=48)** | **Development and external validation (n=14)** |
| --- | --- | --- | --- |
|  | **n (%)** | **n (%)** | **n (%)** |
| **Study characteristics** |  |  |  |
| **Cancer type** |  |  |  |
| Lung | 8 (12.9) | 6 (12.5) | 2 (14.3) |
| Breast | 6 (9.68) | 6 (12.5) | - |
| Colon/colorectal/rectal | 6 (9.68) | 3 (6.3) | 3 (21.4) |
| Pancreatic | 3 (4.84) | 1 (2.1) | 2 (14.3) |
| Liver | 2 (3.23) | 2 (4.2) | - |
| Gastric | 3 (4.84) | 3 (6.3) | - |
| Head and neck | 5 (8.06) | 5 (10.4) | - |
| Spinal | 4 (6.45) | 4 (8.3) | - |
| Brain (inc. meningioma, glioblastoma) | 5 (8.06) | 4 (8.3) | 1 (7.1) |
| Oral (inc. nasopharyngeal carcinoma) | 3 (4.84) | 2 (4.2) | 1 (7.1) |
| Gynaecological (inc. cervical, ovarian, endometrial) | 6 (9.68) | 5 (10.4) | 1 (7.1) |
| Prostate/penile | 5 (8.06) | 4 (8.3) | 1 (7.1) |
| Skin (inc. melanoma) | 2 (3.23) | 1 (2.1) | 1 (7.1) |
| Other* | 4 (6.45) | 2 (4.2) | 2 (14.3) |
| **Target population** |  |  |  |
| Cancer patients | 55 (88.7) | 43 (89.6) | 12 (85.7) |
| Individuals at risk | 1 (1.6) | 1 (2.1) | - |
| General population | 4 (6.5) | 2 (4.2) | 2 (14.3) |
| Sexual minority women | 1 (1.6) | 1 (2.1) | - |
| Unclear | 1 (1.6) | 1 (2.1) | - |
| **Outcome** |  |  |  |
| Binary | 48 (77.4) | 40 (83.3) | 8 (57.1) |
| Continuous | 1 (1.6) | - | 1 (7.1) |
| Multinomial | 2 (3.2) | 2 (4.2) | - |
| Time to event | 11 (17.7) | 6 (12.5) | 5 (35.7) |
| **Development characteristics** |  |  |  |
| **Data source**** |  |  |  |
| Randomised controlled trial | 1 (1.6) | - | 1 (7.1) |
| Prospective cohort | 9 (14.5) | 9 (18.8) | - |
| Retrospective cohort | 14 (22.6) | 11 (22.9) | 3 (21.4) |
| Registry | 21 (33.9) | 15 (31.3) | 6 (42.9) |
| Routine care database | 9 (14.5) | 7 (14.6) | 2 (14.3) |
| Other*** | 3 (4.8) | 2 (4.2) | 1 (7.1) |
| Unclear | 5 (8.1) | 4 (8.3) | 1 (7.1) |
| **Setting****** |  |  |  |
| Primary care | 2 (3.2) | 2 (4.2) | - |
| Secondary care | 36 (58.1) | 29 (60.4) | 7 (50) |
| Tertiary care | 10 (16.1) | 7 (14.6) | 3 (21.4) |
| General population | 5 (8.1) | 3 (6.3) | 2 (14.3) |
| Other***** | 3 (4.8) | 3 (6.3) | - |
| Unclear | 6 (9.7) | 4 (8.3) | 2 (14.3) |
| **Multicentre******** |  |  |  |
| No | 26 (41.9) | 24 (50) | 2 (14.3) |
| Yes | 13 (21) | 7 (14.6) | 6 (42.9) |
| Unclear | 23 (37.1) | 17 (35.4) | 6 (42.9) |
| **Geographic location********* |  |  |  |
| South America | 2 (3.2) | 2 (4.2) | - |
| Asia | 8 (12.9) | 6 (12.5) | 2 (14.3) |
| Europe | 13 (21) | 13 (27.1) | - |
| Canada | 3 (4.8) | 3 (6.3) | - |
| USA | 21 (33.9) | 15 (31.3) | 6 (42.9) |
| Europe, North America, Australia | 1 (1.6) | 1 (2.1) | - |
| Europe, South America | 1 (1.6) | - | 1 (7.1) |
| South Asia, USA | 1 (1.6) | 1 (2.1) | - |
| Unclear | 12 (19.4) | 7 (14.6) | 5 (35.7) |
| **Intended user** |  |  |  |
| Health care providers | 34 (54.8) | 27 (56.3) | 7 (50) |
| Public/patients | 2 (3.2) | 2 (4.2) | - |
| Researchers | 1 (1.6) | 1 (2.1) | - |
| Health care providers and patient/public | 4 (6.5) | 1 (2.1) | 3 (21.4) |
| Health care providers and researchers | 2 (3.2) | 2 (4.2) | - |
| Unclear | 19 (30.6) | 15 (31.3) | 4 (28.6) |
| **Aim of model** |  |  |  |
| Predict risk | 36 (58.1) | 25 (52.1) | 11 (78.6) |
| Classify patients | 25 (40.3) | 23 (47.9) | 2 (14.3) |
| Predict length of stay | 1 (1.6) | - | 1 (7.1) |

*Other includes peritoneal carcinomatosis, incurable cancer (various), leukemia, malignant peripheral nerve sheath tumour

**Validation characteristics for data source are: Randomised controlled trial: 2/14 (14.3%); Prospective cohort:3/14 (21.4%); Retrospective cohort: 4/14 (28.6%); Registry: 2/14 (14.3%); Routine care database: 2/14 (14.3%); Other (survey):1/14 (7.1%)

***Other includes audit, survey and a combination data source of hospital and research data and a registry

****Validation characteristics for setting are: Secondary care: 7/14 (50%); Tertiary care: 4/14 (28.57%); General population: 2/14 (14.29%); Unclear: 1/14 (7.14%).

*****Other includes combination of hospitals, hospices and nursing homes, NTT medical center in Tokyo and combination of primary and tertiary care

******Validation characteristics for multicentre are: No: 8/14 (57.14%); Yes: 3/14 (21.43%); Unclear: 3/14 (21.43%).

*******Validation characteristics for geographical location are: South America: 1/14 (7.14%); Asia: 5/14 (35.71%); USA: 5/14 (35.71%); Unclear: 3/14 (21.43%).

**Supplementary figure 1**. Histogram of the events per predictor, by modelling type. N=65.

**Supplementary figure 2**. Histogram of the events per predictor, by modelling type. Outliers (EPV>4000) are removed. N=58.
